# Response of Growth, Yield, and Phytochemical Behavior of Jojoba Genotypes to *Azolla filiculoides* Plant Extract

**DOI:** 10.3390/plants11101314

**Published:** 2022-05-16

**Authors:** Amira K. G. Atteya, Aishah N. Albalawi, Hala M. Bayomy, Eman S. Alamri, Esmail A. E. Genaidy

**Affiliations:** 1 Horticulture Department, Faculty of Agriculture, Damanhour University, Damanhour 22516, Egypt; 2 Department of Analytical Chemistry, University College of Haql, Tabuk University, Tabuk 71491, Saudi Arabia; 3 Department of Nutrition and Food Science, Tabuk University, Tabuk 71491, Saudi Arabia; hala.biomy@agr.dmu.edu.eg (H.M.B.); ialamri@ut.edu.sa (E.S.A.); 4 Department of Food Science and Technology, Damanhour University, Damanhour 22516, Egypt; 5 Pomology Department, National Research Centre, Giza 12622, Egypt; esmail_nrc@yahoo.com

**Keywords:** jojoba genotypes, growth, yield, fatty acids, gadoleic acid, Azolla extract

## Abstract

A unique storage lipid wax found in jojoba seeds can be employed as a chemical feedstock. Alternative safe and natural sources of plant nutrients are constantly sought to preserve both human health and the environment. As a result, EAI1, EAI2, and EAI3 jojoba genotypes’ growth, yield, and phytochemical behavior in response to Azolla extract at concentrations of 0, 10, and 30% were studied. Maximum productivity was found with EAI1, followed by EAI3 then EAI2 across all Azolla extracts. In relation to the Azolla extract concentrations in the two seasons, the concentration of 30% delivered the most pronounced results across all the studied genotypes. During the two seasons, administration of a combined treatment of the EAI1 jojoba genotype with 30% Azolla extract produced the highest mean values of growth, flowering, and fruiting, as well as chemical composition parameters. This means that the treatment of EAI1 genotype with 30% *Azolla filiculoides* Lam. plant extract yielded the highest significant seed yield (3748 and 3839 kg ha^−1^) and oil yield per hectare (1910 and 2002 L ha^−1^). The combination treatment of the EAI1 jojoba genotype with 10% Azolla extract yielded the highest proportion of gadoleic fatty acid (49.83%).

## 1. Introduction

The Simmondsiaceae family includes the jojoba plant *Simmondsia chinensis* (Link) C.K. Schneid. The Sonoran Desert has become endemic in southern Arizona, southern California, and northern Mexico [1]. A number of dry and semi-arid regions have adopted jojoba shrub farming. Ripe seeds have a hard oval shape, are a dark brown color, and make up the economic portion of the jojoba shrub [2]. Esters derived from acids and alcohols make up the storage lipid wax in jojoba seeds [3]. Jojoba oil is known for its odorless, hygienic, and heat-resistant lubricating properties. As a result, it can be utilized in the chemical industry [1] as a basic feedstock for medications, lubricants, gear additives, extenders, and anti-foaming agents, as well as in the wax and polish industries [3,4]. Plant development, blooming, and fruiting, as well as seed yield and consistency, are influenced by a variety of factors such as environmental conditions, plant growth regulators, nutrients, and genotype [5,6,7,8,9,10]. Plants with increased oil content and more seeds are developed through selective breeding [2]. Genaidy et al. [11] conducted a selective breeding study on jojoba plants and discovered that numerous genotypes of jojoba plants exist in Egypt’s natural habitat. Furthermore, selecting genotypes such as EAI1, EAI2, and EAI3 exhibited nearly optimal values for most variables such as seed production per plant (2756, 2244, and 2402 g plant^−1^) and seed oil percentage (51, 49, and 50%).

Organic extracts are a hot topic in organic agriculture for improving growth performance and yield, modifying phytochemical content [12,13], inducing crop physiology and biochemistry such as photosynthetic rate, enhancing antioxidant machinery [7,14], and improving root system development [15], all of which improve nutrient uptake and enhance efficiency [16]. Fertilizers refer to any natural, synthetic, organic, or inorganic components that are added to soil to provide one or more plant nutrients necessary for plant growth. In income-based agriculture, especially in relation to inorganic fertilizers, the parameter of marginal productivity in production factors use must be considered. Pollution and degradation are a consequence of their misuse. As a result, agricultural farming practices have evolved to become more environmentally friendly, particularly in relation to lowering chemical inputs without lowering agricultural production quantity or quality.

Nitrogen is a macro-element that plays an important function in plant development and growth [17]. Organic and biological sources of nitrogen are an important method for enhancing crop yields under organic farming practices [7,8] because N is required for maximum growth and optimal production. The free-floating fern *Azolla filiculoides* Lam. is native to the American continent [18], and its ability to fix 30–60 kg N ha^−1^ [19,20,21,22] makes it a useful biological nitrogen source. It also has a high mineral content and is abundant in phytohormones (auxins, cytokinins, and gibberellins), essential amino acids, protein, and vitamins [20,23]. As a result, it can be utilized to improve crop yields. Following Azolla treatments, wheat and beet yields were improved [24,25]. The use of Azolla increased the seed yield of quinoa grain by about 29% compared with untreated plants [13] and increased the vegetative growth and biomass of olives in the nursery [26]; eggplant yield was increased from 2837.12 g plant^−1^ for untreated plants to 3600.69 g plant^−1^ for treated plants [27]; squash fruit numbers also increased from 6.67 to 8.00 fruit plant^−1^, and the fruit weight increased from 35.60 to 37.14 g fruit^−1^ [28]. After Azolla supplementation, the amount of urea required for rice and maize production fell by 25% and 30%, respectively [29,30]. Egyptian trade with the European Union is boosted by the implementation of organic agriculture laws for the growth of medicinal and aromatic plants [31]. To our knowledge, no research has been conducted on the effects of jojoba genotypes and *Azolla filiculoides* Lam. extract on jojoba plant development. The purpose of this study was to determine how different jojoba genotypes and *Azolla filiculoides* Lam. extracts, as well as combinations of the two, influenced the growth, yield, and chemical composition of a jojoba shrub grown in an organic farming system.

## 2. Results

### 2.1. Growth Parameters

**Figure 1:**
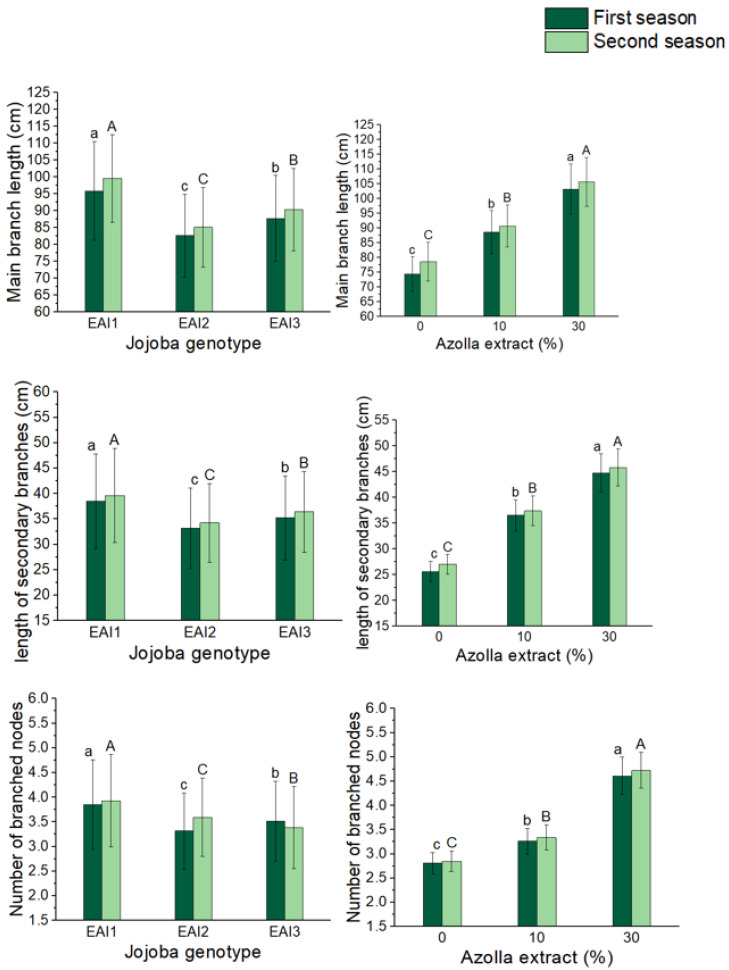
In both seasons, the mean values of main branch length (cm), secondary branch length (cm), and the number of branched nodes of jojoba shrubs were modified by plant genotypes and foliar application of *Azolla filiculoides* Lam. plant extract respectively. The data are expressed as a mean value ± SE. At the 0.05 level, bars with identical letters are not significant.

**Figure 2:**
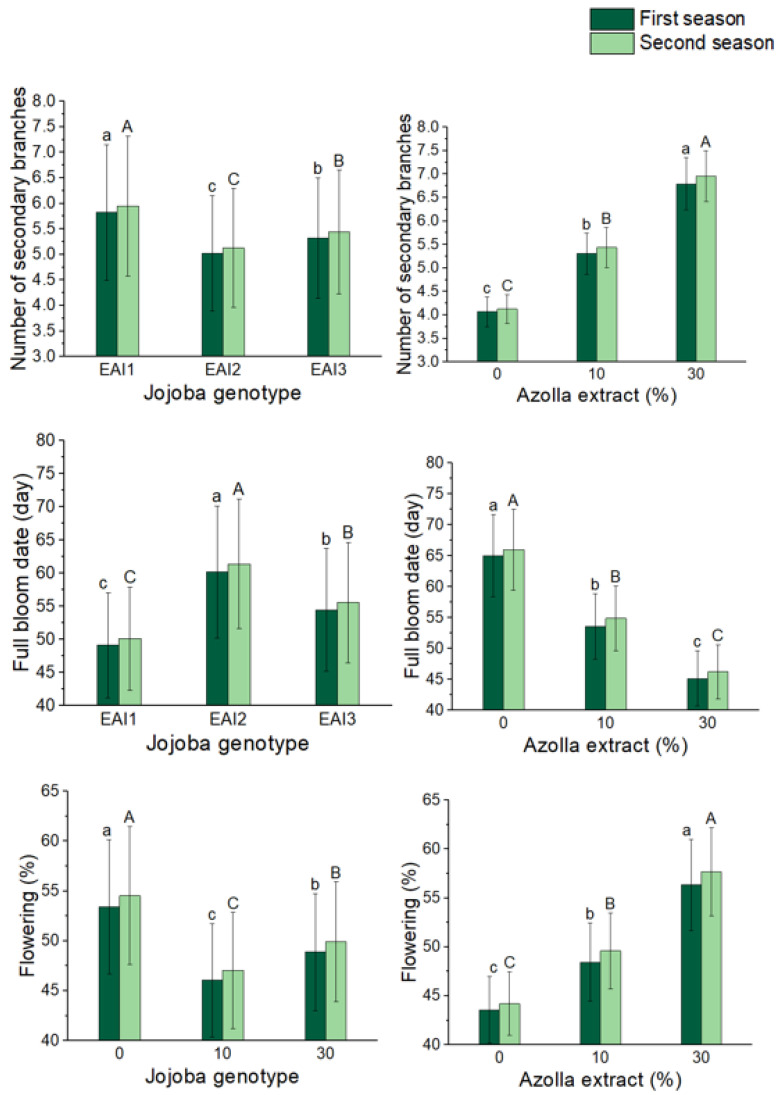
In both seasons, the mean values of the number of secondary branches, full bloom date (day), and flowering percentage (%) of jojoba shrubs were modified by plant genotypes and foliar application of *Azolla filiculoides* Lam. plant extract respectively. The data are expressed as a mean value ± SE. At the 0.05 level, bars with identical letters are not significant.

The vegetative features of jojoba shrubs were considerably altered by plant genotypes and the foliar treatment of *Azolla filiculoides* Lam. plant extract. The genotype EAI1 of jojoba shrubs had the highest mean values of main branch length (95.85 and 99.55 cm), secondary branch length (38.51 and 39.63 cm), number of branched nodes (3.85 and 3.93), and number of secondary branches (5.82 and 5.95) in both seasons, according to Table 1 and Table 2 and Figure 1 and Figure 2. When comparing spraying levels of *Azolla filiculoides* Lam. plant extract, a 30% application resulted in the most significant mean values of shrub growth parameters, with increases of 39 and 34% for main branch length, 75 and 70% for secondary branch length, 64 and 66% for the number of branched nodes, and 67 and 69% for the number of secondary branches, when compared to the control shrubs. Plant genotypes and foliar application of *Azolla filiculoides* Lam. plant extract had significant interaction. Furthermore, the maximum significant mean values of main branch length (111.8 and 114.5 cm), secondary branch length (48.5 and 49.6 cm), number of branched nodes (4.99 and 5.11), and number of secondary branches (7.35 and 7.53) were recorded with the combined treatment of the EAI1 jojoba genotype with 30%% *Azolla filiculoides* Lam. plant extract in both seasons.

### 2.2. Flowering and Fruit Set Parameters

**Figure 3:**
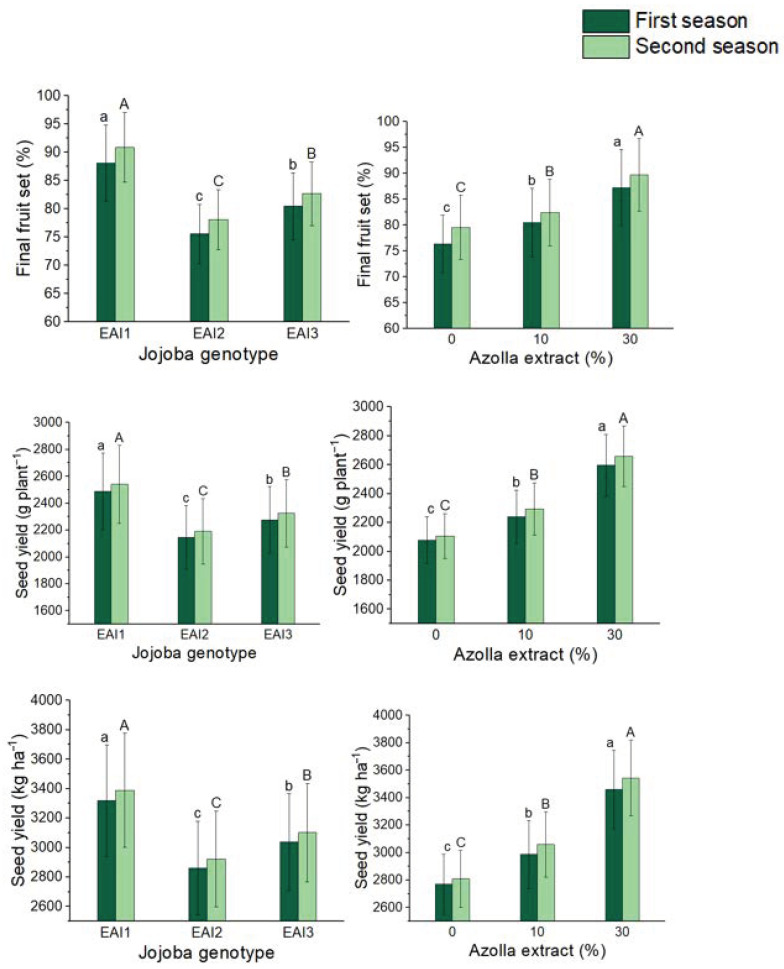
In both seasons, the mean values of final fruit set (%), seed yield (g plant^−1^), and seed yield (kg ha^−1^) of jojoba shrubs were modified by plant genotypes and foliar application of *Azolla filiculoides* Lam. plant extract respectively. The data are expressed as a mean value fruit set (%), seed yield (g) planting and fruit set parameters in both seasons. At the 0.05 level, bars with identical letters are not significant.

Figure 2 and Figure 3 as well as Table 2 and Table 3 reveal that jojoba genotypes, the foliar application of *Azolla filiculoides* Lam. plant extract, and their combined treatments dramatically improved all blooming and fruiting metrics. For the first and second seasons, the earliest full bloom dates were 49 and 50 days, respectively; maximum flowering percentages of 53 and 55% and final fruit set percentages of 88 and 91% were found with the jojoba genotype of EAI1 in the first and second seasons, respectively. When compared to other extract concentrations and controls, flowering and fruit set characteristics were dramatically improved when employing 30% *Azolla filiculoides* Lam. plant extract. In comparison to control shrubs, the reduction in full bloom date was around 20 days for both seasons; the increase in flowering percentage was 29 and 31%, and the final fruit set percentage was 14 and 13% for the first and second seasons, respectively. In terms of combined treatments, the combination treatment of the EAI1 genotype with 30% *Azolla filiculoides* Lam. plant extract produced the greatest significant increases in flowering and fruit set parameters in both seasons. It also had the earliest full bloom date (40.78 and 41.76 days), as well as the highest blooming percentage (61.01 and 62.49%) and final fruit set percentage (94.8 and 97.1%).

### 2.3. Seed Yield

With the treatment of both jojoba genotypes, the foliar application of *Azolla filiculoides* Lam. plant extract and seed output per tree and per hectare of jojoba bushes have been dramatically altered (Table 3). In both seasons, EAI1 jojoba genotype seeds produced the highest seed yield per bush (2489 and 2541 g plant^−1^) and per hectare of jojoba shrubs (3318 and 3389 kg ha^−1^). In addition, foliar treatment of 30% increased seed yield per tree (25 and 26%) and per hectare (25 and 26%), which was the most when compared to the control. Similarly, in both seasons, treatment of the EAI1 genotype with 30% *Azolla filiculoides* Lam. plant extract yielded the highest significant seed yield per tree (2811 and 2879 g plant^−1^) and per hectare (3748 and 3839 kg ha^−1^).

### 2.4. Seed Chemical Compounds

**Figure 4:**
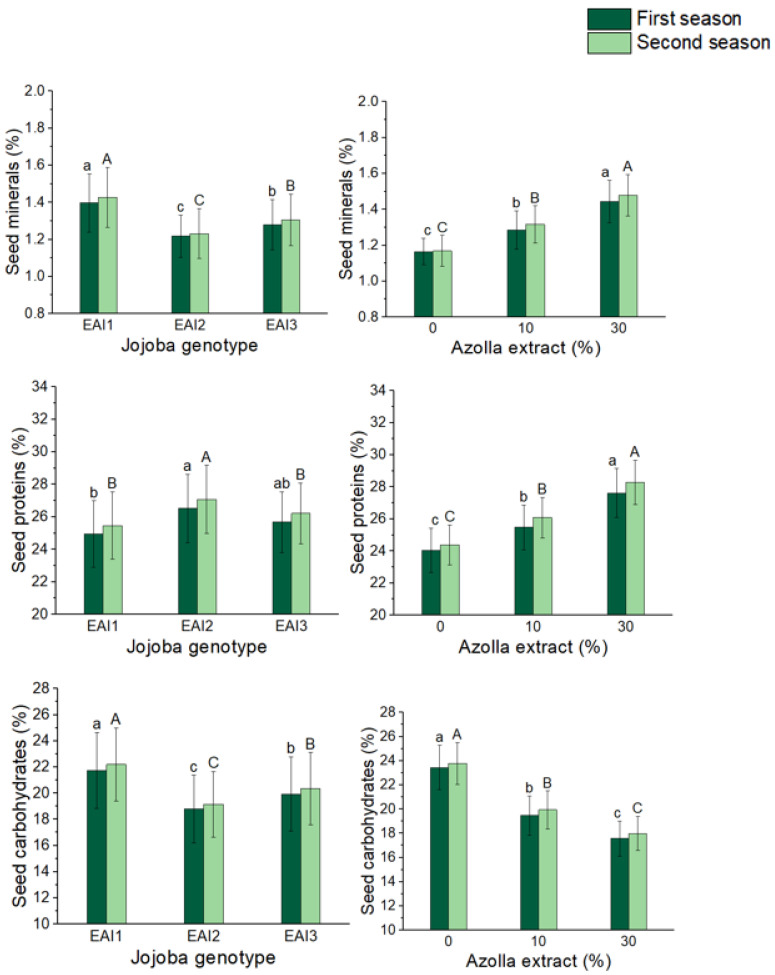
In both seasons, the mean values of minerals (%), proteins (%), and carbohydrates (%) of jojoba shrubs were modified by plant genotypes and foliar application of *Azolla filiculoides* Lam. plant extract respectively. The data are expressed as a mean value ± SE. At the 0.05 level, bars with identical letters are not significant.

**Figure 5:**
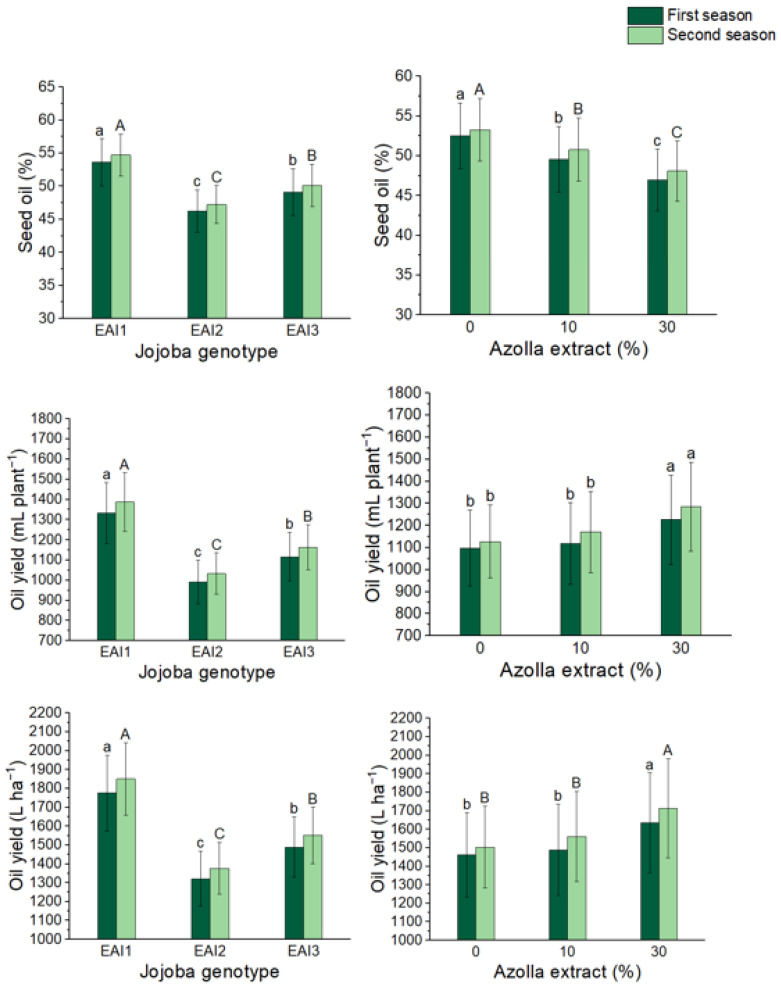
In both seasons, the mean values of oil percent (%), oil content (mL plant^−1^), and oil yield (L ha^−1^) of jojoba shrubs were modified by plant genotypes and foliar application of *Azolla filiculoides* Lam. plant extract respectively. The data are expressed as a mean value ± SE. At the 0.05 level, bars with identical letters are not significant.

The effect of jojoba genotypes and foliar application of *Azolla filiculoides* Lam. plant extract rates on jojoba shrub seed chemical components are shown in Table 4 and Table 5. Minerals (1.40 and 1.43%), fixed oil (53.61 and 54.73%), and carbohydrate percentages were all significantly higher in the EAI1 genotype (21.75 and 22.19%) (Figure 4 and Figure 5). For the application of *Azolla filiculoides* Lam. plant extract, the foliar treatment of 30% *Azolla filiculoides* Lam. plant extract had the most substantial increase in minerals (24 and 26%) and proteins (26.52 and 27.08%) (15 and 16%). In the first and second seasons, the control shrubs had the highest fixed oil percentage (52.50 and 53.26%) and carbohydrates percentage in the jojoba seeds (23.43 and 23.77%), respectively. In all the seasons, the combined treatments had an effect on minerals, proteins, and fixed oil percentage in jojoba seeds. Furthermore, treatment of the EAI1 genotype with 30% *Azolla filiculoides* Lam. plant extract resulted in significant maximum mean values of minerals (1.56 and1.60%), and treatment of the EAI2 genotype with 30% *Azolla filiculoides* Lam. plant extract resulted in significant maximum mean values of protein percentage (1.56 and 1.60%) (28.52 and 29.20%). With the combination treatment of the EAI1 genotype with 0% *Azolla filiculoides* Lam. plant extract, the maximum mean value of carbohydrates (25.14 and 25.50%) and fixed oil percentage (56.3 and 57.1%) were reported.

### 2.5. Yield of Seed Oil 

The impact of plant genotypes and foliar application of *Azolla filiculoides* Lam. plant extract on jojoba seed oil content per shrub and production per hectare is shown in Figure 5 and Table 5. The EAI1 genotype exhibited the highest oil content in jojoba seeds per shrub (1332 and 1387 mL plant^−1^) as well as the highest production per hectare (1776 and 1850 L ha^−1^). The use of a 30% extract of an *Azolla filiculoides* Lam. plant resulted in the highest jojoba seed oil content per bush and output per hectare. In the first and second seasons, it boosted seed oil content per shrub and yield per hectare by 12 and 14%, respectively in both seasons as compared to non-foliar bushes. In the combination treatments, the EAI1 genotype with 30% *Azolla filiculoides* Lam. plant extract produced the highest significant jojoba seed oil content per shrub (1432 and 1501 mL plant^−1^) and yield per hectare (1910 and 2002 L ha^−1^) in both seasons.

### 2.6. Seed Fixed Oil Analysis

Table 6 shows that the fixed oil of jojoba seeds is high in unsaturated fatty acids, with values ranging from 78.05 to 93.98% for the EAI2 genotype with 0% *Azolla filiculoides* Lam. plant extract and the EAI1 genotype with 10% *Azolla filiculoides* Lam. plant extract treatments. For the EAI2 genotype with 0% *Azolla filiculoides* Lam. plant extract and the EAI1 genotype with 10% *Azolla filiculoides* Lam. plant extract, the total known components ranged from 81.38 to 96.15%, respectively. Gadoleic acid, oleic acid, erucic acid, and nervonic acid are the primary fatty acids found in jojoba oil. The major fatty acids in jojoba oil are gadoleic acid, which ranges from 43.11 to 49.83%, oleic acid, which ranges from 10.11 to 13.61%, and erucic acid, which ranges from 12.15 to 14.97% for the combined treatment of the EAI2 genotype with 0% *Azolla filiculoides* Lam. plant extract and combined treatment of the EAI1 genotype with 10% *Azolla filiculoides* Lam. plant extract, respectively. The proportion of nervonic acid in the combined treatment of the EAI2 genotype with 10% *Azolla filiculoides* Lam. plant extract and combined treatment of the EAI1 genotype with 30% *Azolla filiculoides* Lam. plant extract was 11.07 and 12.96%, respectively.

### 2.7. Chlorophyll a and b

**Figure 6:**
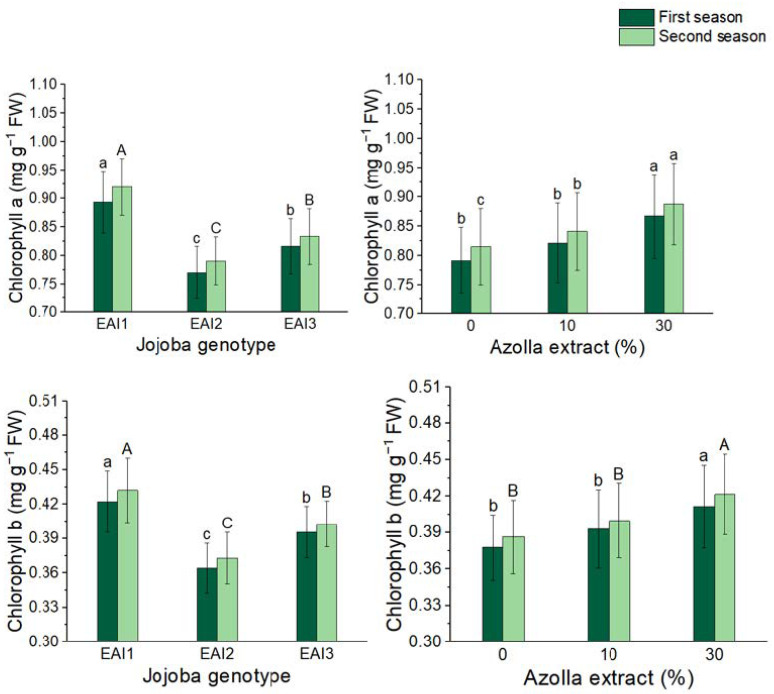
In both seasons, the mean values of chlorophyll a and b (mg g−1 FW) of jojoba shrubs were modified by plant genotypes and foliar application of *Azolla filiculoides* Lam. plant extract respectively. The data are expressed as a mean value ± SE. at the 0.05 level, bars with identical letters are not significant.

All plant genotype treatments, as well as the foliar application of *Azolla filiculoides* Lam. plant extract, resulted in significant increases in chlorophyll a and b of jojoba leaves in both seasons (Figure 6 and Table 7). Chlorophyll a and b levels increased significantly in the EAI1 genotype, with the highest mean chlorophyll a (0.867 and 0.888 mg g^−1^ FW) and chlorophyll b (0.867 and 0.888 mg g^−1^ FW) values (0.412 and 0.422 mg g^−1^ FW). Chlorophyll a increased by 10% and 9% in shrubs sprayed with 30% *Azolla filiculoides* Lam. plant extract in both seasons, whereas chlorophyll b increased by 13 and 11% compared to untreated bushes. The highest mean chlorophyll a (0.939 and 0.962 mg g^−1^ FW) and b (0.446 and 0.457 mg g^−1^ FW) values were obtained in the two seasons when the EAI1 genotype was coupled with the highest rate of *Azolla filiculoides* Lam. plant extract (30%) (Table 7).

### 2.8. Macro Elements (N, P, and K)

**Figure 7:**
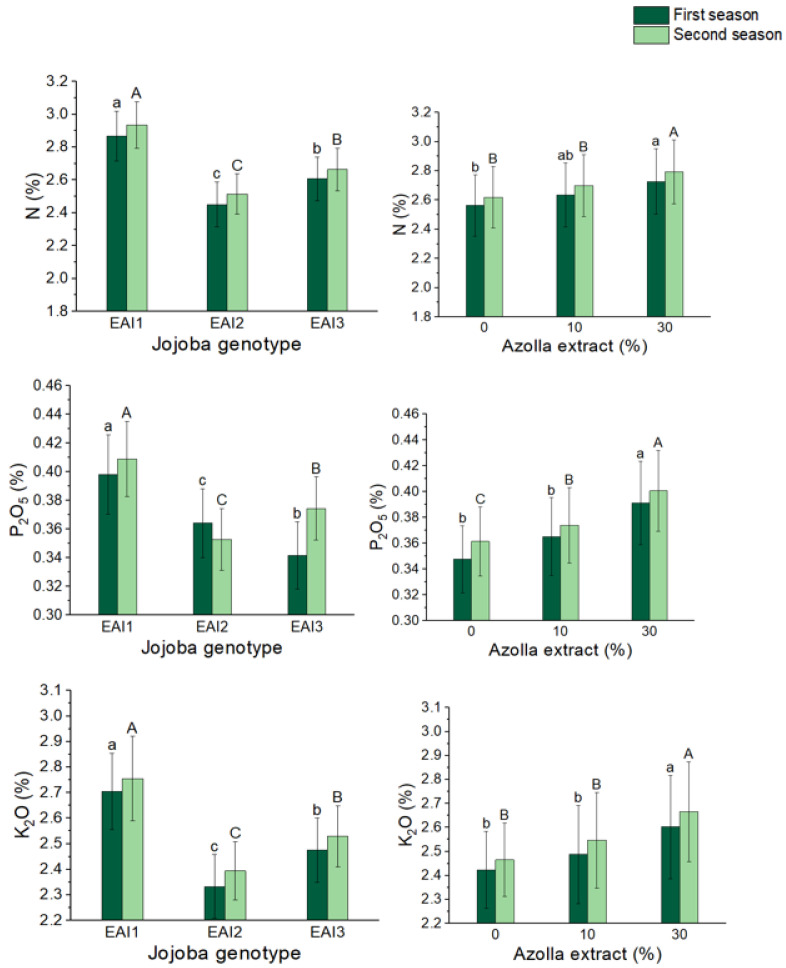
In both seasons, the mean values of nitrogen (%), phosphorus (%), and potassium percentage (%) of jojoba shrubs were modified by plant genotypes and foliar application of *Azolla filiculoides* Lam. plant extract respectively. The data are expressed as a mean value ± SE. at the 0.05 level, bars with identical letters are not significant.

The findings of nitrogen, phosphate, and potassium assays in jojoba leaves are shown in Figure 7 and Table 8. Plant genotype had a considerable impact on the nitrogen, phosphate, and potassium content of jojoba shrub leaves. In both seasons, EAI1 jojoba shrubs had the greatest mean nitrogen (2.87 and 2.94%), phosphorus (0.398 and 0.409%), and potassium percentage (2.70 and 2.75%). After the foliar application of *Azolla filiculoides* Lam. plant extract, the proportion of N, P, and K in jojoba leaves increased significantly. When compared to control plants, the 30% *Azolla filiculoides* Lam. plant extract resulted in the biggest increases in mean percentages of the nitrogen (6 and 7%), phosphorus (13 and 11%), and potassium (7 and 8%) in jojoba leaves. Similarly, in both seasons, the combined treatment of the EAI1 genotype with 30% *Azolla filiculoides* Lam resulted in the greatest significant mean nitrogen (2.95 and 3.03%), phosphorus (0.424 and 0.434%), and potassium (2.82 and 2.89%) concentrations in jojoba leaves.

## 3. Discussion

### 3.1. Effect of Jojoba Genotypes

When the three jojoba genotypes were tested in this experiment, it was discovered that the shrubs of the EAI1 genotype outperformed in every studied parameter, including growth, flowering, fruit set, chlorophyll a and b content, and N, P, and K percentages. The differences in vegetative features between genotypes could be explained by natural growth habits and genotype branching [32,33,34]. Depending on its genetic makeup, each genotype has its own chemical composition. As a result, distinct genomic expressions were expected to be detected in the chemical parameters studied [35]. Al-Soqeer et al. [36] concur that there are variances amongst jojoba plants. Our findings are consistent with those of Al-Soqeer et al. [34], who assessed the development and yield of seven genotypes planted at Qassim University Farm. They found that the genotypes differed significantly for all of the traits tested. HB2 had the most branching nodes, total nodes, flower buds and flowers, total leaves per branch, and branch length and internodes while having the shortest branch length and internodes. In terms of branch length, branch dry weight, longest internodes length, seed yield plant^−1^, and seed weight, genotype HB8 was superior. Flowering initiation differences were also discovered among the seven genotypes studied. For plants that were roughly five years old, the seed yield per plant ranged from 335.3 to 821.6 g plant^−1^. Through an evaluation of the oil seed percentage of jojoba shrubs, Al-Soqeer et al. [36] found differences between genotypes which ranged between 47.17 and 54.95%. Eltaweel et al. [37] recorded that vegetative and flowering chlorophyll a and b differed according to genotype, and the seed yield ranged between 1 and 3.5 kg shrub^−1^ and the oil percentage of seed ranged between 49.50 and 61.17% for eight years old plants. Nahla et al. [38] found that the performance of cultivated plants of jojoba shrubs differed as a result of high variability within plant genotypes. Moreover, the seed yield ranged between 1.145 and 3.650 kg plant^−1^.

### 3.2. Effect of Azolla Filiculoides Extract

Growth, flowering, fruit set, chlorophyll a and b content, and N, P, and K percentages all improved as the extract concentration of the *Azolla filiculoides* Lam. plants increased. This could be due to the combined actions of macro and micronutrients, amino acids, vitamins, ascorbates, phenolic compounds antioxidants, and growth-regulating hormones contained in the *Azolla filiculoides* Lam. plant extract such as auxins, cytokinins, gibberellins, jasmonic, and salicylic acids [20]. In this study, the improvement in vegetative and flowering yields was matched in the jojoba shrub’s seed production and oil output. Fruits are the primary source of plant yield. Gibberellins boost sink demand by elongating fruit cells and improving phloem unloading and/or carbon assimilation and metabolism in fruit. Gibberellins cause sugar metabolizing enzymes to become active. As a result, seed and oil yields are increased [6,7,39,40,41]. Our findings are consistent with the findings of a number of other scholars. *Azolla filiculoides* Lam. plant extract has been shown to boost the growth, yield, and chemical composition of a variety of crops, including the vegetative growth and biomass of olive seedlings [26], squash fruit numbers, which were 19.49% and 4.33% per plant and fruit weight, respectively [28], eggplant yield, which was 26.91% [27], and quinoa grain yield, which was 29% [13]. Increases in *Azolla filiculoides* Lam. plant extract, on the other hand, reduced the percentages of jojoba fixed oil and carbs in both seasons of this study. Plant metabolism changes towards the formation of N-compounds in high N circumstances, with less production of secondary non-N-containing metabolites such as phenolics, fatty acids, and terpenoids [42,43]. These results are similar to those of Atteya et al. [8,9].

### 3.3. Effect of Combination Treatments

Each genotype under investigation has a unique genetic profile [11]. Depending on the genotype, each genetic character’s reactions change with climatic and soil circumstances [34,36,40,41]. The growth and outputs of jojoba genotypes improved after foliar spraying with *Azolla filiculoides*, however, the degree of response was different for each genotype. *Azolla filiculoides* is widely used in agriculture to improve plant development [26,27,28] and plant tolerance to biotic and abiotic stress [30,44,45]. Azolla is a plant that fixes biological nitrogen and reduces fertilizer leaching [46]. Previous studies have investigated using Azolla as a compost or soil supplement in green manure to boost crop output. However, there is limited information about using Azolla extract as a foliar spray, especially in organic farming. Azolla is high in macro- and micronutrients, crude protein, growth-promoting cytokinins, jasmonic acid, and salicylic acid, as well as vitamins [18,46]. This could increase jojoba genotypes’ plant growth and development by increasing endogenous phytohormone levels and nutrient uptake. Azolla phytohormones known as cytokinins induce cell division and change apical dominance [13]. As a result of the Azolla supplementation, we saw an improvement in the development and yield attributes of all of the jojoba genotypes examined. These findings are consistent with those of Ripley et al. [24], who demonstrated improvement among wheat plants treated with *Azolla filiculoides*, and that the mean grain yield per plant increased by 47.06% compared with the control group. Eggplant fruit yield was significantly raised with the addition of Azolla by 26.91% [27]. After foliar spraying with Azolla, the total chlorophyll in jojoba genotypes increased. The necessary components for chlorophyll production, N and Mg, are abundant in Azolla. Furthermore, the increase in photosynthetic pigments after the Azolla spray could be attributed to Azolla’s chlorophyll and carotenoids. The chlorophyll content of Beta vulgaris [25] and quinoa [13] was boosted by the foliar application of *Azolla filiculoides* compost by 300% and 19.7%, respectively. In squash, dry Azolla dramatically boosted leaf colors by about 41%, improved fruit characteristics by 19.49% and 4.33% for fruit number per plant and fruit weight, respectively, and increased nutrient content by about 60% [28]. The percentage of fixed oil and major fatty acids with the results of jojoba seeds contain approximately 53.68% fixed oil, according to Atteya et al. [6,7], with the major fatty acid gadoleic acid accounting for 53.54% and oleic acid contributing 15.59%. The EAI1, EAI2, and EAI3 jojoba shrub genotypes are distinguished by high seed and oil yield, according to Genaidy et al. [11].

## 4. Materials and Methods

The effects of jojoba genotypes and *Azolla filiculoides* Lam. plant extract, as well as their combinations, on the growth, yield, and chemical composition of a jojoba shrub grown under an organic farming approach were studied at a private farm in Egypt’s El-Beheira governorate. Jojoba shrubs of equivalent vigor, age (twelve years old), and size were chosen for the administration of the investigated treatments across the two study seasons (2019/2020 and 2020/2021). In a randomized complete block design, the experiment was set up as a factorial with three replications (RCBD). For each jojoba genotype, the same shoot was chosen for each treatment in terms of height, thickness, vigor, amount of fruit, and orientation (EAI1, EAI2, and EAI3). The spacing between rows and between bushes in the rows was 3 and 2.5 m, respectively. A drip irrigation system was employed in the orchard. All of the bushes in the study were subjected to identical conditions, such as irrigation and farming practices. The orchard soil analysis was performed at the beginning of the experiment (Table 9) and the results of the water irrigation analysis are reported in Table 10.

### 4.1. Azolla Filiculoides *Lam.* Plant Extract Preparation

*Azolla filiculoides* Lam. aqueous extract solutions were prepared by drying the plant material in an oven at 70 °C for 48 h. The dry material was crushed in a grinder and filtered through a 40 mesh screen. To make the extracts, 300 g of each crushed plant material was macerated in 1000 mL of distilled water. Solutions were shaken in an orbital shaker at room temperature for 24 h. Whatman filter paper no. 1 was used to filter the extracts. To attain the concentrations, the extracted extracts were diluted. Table 11 shows the results of various analyses of the *Azolla filiculoides* Lam. plant extract study. The treatments carried out using this extract are provided in Table 12.

### 4.2. Treatments

This study used three jojoba genotypes (EAI1, EAI2, and EAI3) [11] to study the response of the jojoba plant to the application of *Azolla filiculoides* Lam plant extract application. In each experimental year, from the beginning of December, 4 L of *Azolla filiculoides* Lam. plant extract was sprayed on each tree every 2 weeks (17 applications shrub^−1^). Treatments for *Azolla filiculoides* Lam. included 0, 10, and 30% plant extract.

### 4.3. Parameters

Nine plants were randomly chosen and labeled for each treatment (three bushes for each replicate).

#### 4.3.1. Branch Characters 

Branch length (cm), length of secondary branches per every branch (cm), number of nodes forming branches per meter, and number of secondary branches per main branch per meter.

#### 4.3.2. Flowering, Fruiting, and Seed Yield 

Three branches per tree from each treatment were tagged in December 2019 and 2020, and the full bloom date, flowering percentage, final fruit set percentage, and seed yield (g tree^−1^ and kg ha^−1^) were recorded as mentioned by Atteya et al. [7]. 

#### 4.3.3. Chemical Analyses of Seed

The Soxhlet method was used to extract jojoba oil from seeds, and the oil percentage (%) and yield per hectare were calculated (L ha^−1^). Crude protein (%), mineral (%), and total carbs (%) were also measured, according to the AOAC [47]. Seed fixed oil tests were carried out, in accordance with Atteya et al. [6].

#### 4.3.4. Chlorophyll a and b 

Chlorophyll a and b Were Determined According to Wintermans and Mats [48]. 

#### 4.3.5. The Macro Elements (N, P, and K)

The Method of Chapman and Pratt [49] Was Used to Determine the Amount of Macro Elements in the leaVes.

### 4.4. Statistical Analysis 

SAS software was used to perform an analysis of variance on the test treatment data [50]. At a 5% level of probability, the Duncan test was performed to compare the means of the treatments. Data refers to the mean value SE in the first and second seasons in the Tables.

## 5. Conclusions

The goal of this study was to learn more about the potential benefits of *Azolla filiculoides* Lam. plant extract in boosting plant growth, yield, seed quality, and the phytochemical content of three jojoba shrub genotypes in organic agriculture. The yield of the three jojoba genotypes was significantly influenced by foliar spraying with *Azolla filiculoides* Lam. plant extract. In terms of the qualities described, the EAI1 genotype outperformed the EAI2 and EAI3 genotypes. The application of 30% *Azolla filiculoides* Lam. plant extract was more successful in increasing jojoba shrub yields. Under organic farming settings, the EAI1 genotype exposed to the foliar treatment of *Azolla filiculoides* Lam. plant extract at a rate of 30% generated maximum seed output with the highest fixed oil yield.

## Figures and Tables

**Table 1:** In both seasons of the study, the mean values of main branch length (cm), secondary branch length (cm), and the number of branched nodes of jojoba shrubs as influenced by combined treatments of plant genotypes and foliar application of *Azolla filiculoides* Lam. plant extract.

Treatment		Main Branch Length (cm)		Length of Secondary Branches (cm)		Number of Branched Nodes	
Jojoba Genotype	Azola Extract (%)	1st Season	2nd Season	1st Season	2nd Season	1st Season	2nd Season
EAI1	Control	79.9 ± 4.5 de	85.9 ± 2.7 c	27.5 ± 1.6 e	28.7 ± 1.4 e	3.01 ± 0.17 de	3.06 ± 0.14 ef
EAI1	10	95.9 ± 5.4 b	98.3 ± 4.6 b	39.6 ± 2.2 c	40.5 ± 1.9 c	3.53 ± 0.20 c	3.62 ± 0.17 d
EAI1	30	111.8 ± 6.3 a	114.5 ± 5.4 a	48.5 ± 2.7 a	49.6 ± 2.3 a	4.99 ± 0.28 a	5.11 ± 0.24 a
EAI2	Control	69.4 ± 3.9 f	72.5 ± 3.4 d	23.9 ± 1.3 f	25.2 ± 1.2 f	2.62 ± 0.15 f	2.65 ± 0.13 g
EAI2	10	82.5 ± 4.7 cd	84.5 ± 4.0 c	34.0 ± 1.9 d	34.8 ± 1.6 d	3.04 ± 0.17 de	3.11 ± 0.15 e
EAI2	30	96.1 ± 5.4 b	98.4 ± 4.7 b	41.7 ± 2.4 bc	42.7 ± 2.0 bc	4.29 ± 0.24 b	4.40 ± 0.21 c
EAI3	Control	74.1 ± 4.2 ef	77.4 ± 3.7 d	25.5 ± 1.4 ef	27.2 ± 1.3 ef	2.80 ± 0.16 ef	2.84 ± 0.13 fg
EAI3	10	87.3 ± 4.9 c	89.4 ± 4.2 c	36.0 ± 2.0 d	36.9 ± 1.7 d	3.21 ± 0.18 d	3.29 ± 0.16 e
EAI3	30	101.7 ± 5.7 b	104.2 ± 4.9 b	44.1 ± 2.5 b	45.2 ± 2.1 b	4.54 ± 0.26 b	4.65 ± 0.22 b
F value		49.50 **	59.20 **	107.13 **	139.18 **	103.63 **	149.51 **

The data are expressed as a mean value ± SE. At the 0.05 significance level, the means in columns with the same letter are not significantly different. (**) It means highly significant differences between means in the same column.

**Table 2:** In both seasons of the study, the mean values of the number of secondary branches, full bloom date (day), and flowering percentage (%) of jojoba shrubs as influenced by combined treatments of plant genotypes and foliar application of *Azolla filiculoides* Lam. plant extract.

Treatment		Number of Secondary Branches		Full Bloom Date (Day)		Flowering Percentage (%)	
Jojoba Genotype	Azola Extract (%)	1st Season	2nd Season	1st Season	2nd Season	1st Season	2nd Season
EAI1	Control	4.36 ± 1.36 e	4.43 ± 0.21 e	58.19 ± 3.28 c	59.02 ± 2.79 c	46.76 ± 2.64 cd	47.42 ± 2.24 c
EAI1	10	5.75 ± 1.92 c	5.89 ± 0.28 c	48.42 ± 2.73 ef	49.59 ± 2.35 ef	52.46 ± 2.96 b	53.73 ± 2.54 b
EAI1	30	7.35 ± 2.35 a	7.53 ± 0.36 a	40.78 ± 2.30 g	41.76 ± 1.98 g	61.01 ± 3.44 a	62.49 ± 2.96 a
EAI2	Control	3.79 ± 1.19 f	3.84 ± 0.18 f	71.68 ± 4.05 a	72.71 ± 3.44 a	40.60 ± 2.29 e	41.18 ± 1.95 e
EAI2	10	4.95 ± 1.65 d	5.06 ± 0.24 d	59.08 ± 3.34 c	60.50 ± 2.86 c	45.11 ± 2.55 cd	46.20 ± 2.19 cd
EAI2	30	6.32 ± 2.02 b	6.48 ± 0.31 b	49.75 ± 2.81 de	50.95 ± 2.41 de	52.47 ± 2.96 b	53.74 ± 2.54 b
EAI3	Control	4.05 ± 1.29 ef	4.11 ± 0.19 ef	65.25 ± 3.68 b	66.19 ± 3.13 b	43.38 ± 2.45 de	44.00 ± 2.08 de
EAI3	10	5.23 ± 1.74 d	5.36 ± 0.25 d	53.26 ± 3.01 d	54.55 ± 2.58 d	47.74 ± 2.69 c	48.89 ± 2.31 c
EAI3	30	6.69 ± 2.14 b	6.85 ± 0.32 b	44.85 ± 2.53 fg	45.94 ± 2.17 f	55.52 ± 3.13 b	56.86 ± 2.69 b
F value		103.63 **	149.51 **	64.21 **	86.61 **	36.60 **	53.01 **

The data are expressed as a mean value ± SE. At the 0.05 significance level, the means in columns with the same letter are not significantly different. (**) It means highly significant differences between means in the same column.

**Table 3:** In both seasons of the study, the mean values of final fruit set (%), seed yield (g plant^−1^), and seed yield (kg ha^−1^) of jojoba shrubs as influenced by combined treatments of plant genotypes and foliar application of *Azolla filiculoides* Lam. plant extract.

Treatment		Final Fruit Set (%)		Seed Yield (g plant^−1^)		Seed Yield (kg ha^−1^)	
Jojoba Genotype	Azola Extract (%)	1st Season	2nd Season	1st Season	2nd Season	1st Season	2nd Season
EAI1	Control	82.3 ± 3.1 bc	86.2 ± 4.1 bcd	2229 ± 126 c	2260 ± 107 c	2972 ± 168 c	3014 ± 143 c
EAI1	10	87.2 ± 4.9 b	89.3 ± 4.2 b	2426 ± 137 b	2485 ± 118 b	3235 ± 183 b	3313 ± 157 b
EAI1	30	94.8 ± 5.4 a	97.1 ± 4.6 a	2811 ± 159 a	2879 ± 136 a	3748 ± 212 a	3839 ± 182 a
EAI2	Control	71.0 ± 2.7 e	74.1 ± 3.5 g	1935 ± 109 d	1963 ± 93 e	2580 ± 146 d	2617 ± 124 e
EAI2	10	75.0 ± 4.2 de	76.8 ± 3.6 fg	2086 ± 118 cd	2137 ± 101 cd	2782 ± 157 cd	2849 ± 135 cd
EAI2	30	80.7 ± 3.6 bcd	83.5 ± 4.0 cde	2417 ± 136 b	2476 ± 117 b	3223 ± 182 b	3301 ± 156 b
EAI3	Control	75.8 ± 3.4 de	78.4 ± 3.7 efg	2068 ± 117 cd	2097 ± 99 de	2757 ± 156 cd	2797 ± 132 de
EAI3	10	79.3 ± 4.5 cd	81.2 ± 3.8 def	2208 ± 125 c	2261 ± 107 c	2944 ± 166 c	3015 ± 143 c
EAI3	30	86.3 ± 4.9 b	88.4 ± 4.2 bc	2558 ± 144 b	2620 ± 124 b	3411 ± 193 b	3493 ± 165 b
F value		17.28 **	24.46 **	31.29 **	45.14 **	31.29 **	45.14 **

The data are expressed as a mean value ± SE. At the 0.05 significance level, the means in columns with the same letter are not significantly different. (**) It means highly significant differences between means in the same column.

**Table 4:** In both seasons of the study, the mean values of minerals (%), protein (%), and carbohydrates (%) of jojoba shrubs as influenced by combined treatments of plant genotypes and foliar application of *Azolla filiculoides* Lam. plant extract.

Treatment		Minerals (%)		Proteins (%)		Carbohydrates (%)	
Jojoba Genotype	Azola Extract (%)	1st Season	2nd Season	1st Season	2nd Season	1st Season	2nd Season
EAI1	Control	1.24 ± 0.07 cde	1.25 ± 0.06 d	23.12 ± 1.31 f	23.45 ± 1.11 f	25.14 ± 1.42 a	25.50 ± 1.21 a
EAI1	10	1.39 ± 0.08 b	1.43 ± 0.07 b	24.82 ± 1.40 def	25.42 ± 1.20 de	21.09 ± 1.19 c	21.60 ± 1.02 c
EAI1	30	1.56 ± 0.09 a	1.60 ± 0.08 a	26.90 ± 1.52 abc	27.55 ± 1.30 abc	19.02 ± 1.07 d	19.47 ± 0.92 d
EAI2	Control	1.11 ± 0.02 f	1.09 ± 0.05 f	24.74 ± 1.40 def	25.10 ± 1.19 ef	21.83 ± 1.23 bc	22.15 ± 1.05 c
EAI2	10	1.20 ± 0.07 def	1.23 ± 0.06 de	26.31 ± 1.49 bcd	26.94 ± 1.27 bcd	18.13 ± 1.02 de	18.57 ± 0.88 de
EAI2	30	1.34 ± 0.08 bc	1.38 ± 0.07 bc	28.52 ± 1.61 a	29.20 ± 1.38 a	16.35 ± 0.92 f	16.75 ± 0.79 f
EAI3	Control	1.15 ± 0.06 ef	1.16 ± 0.06 ef	24.28 ± 1.37 ef	24.63 ± 1.17 ef	23.33 ± 1.32 b	23.66 ± 1.12 b
EAI3	10	1.27 ± 0.07 cd	1.30 ± 0.06 cd	25.31 ± 1.43 cde	25.92 ± 1.23 cde	19.19 ± 1.08 d	19.65 ± 0.93 d
EAI3	30	1.42 ± 0.08 b	1.46 ± 0.07 b	27.44 ± 1.55 ab	28.10 ± 1.33 ab	17.30 ± 0.98 ef	17.72 ± 0.84 ef
F value		20.70 **	44.26 **	11.94 **	17.00 **	43.49 **	57.37 **

The data are expressed as a mean value ± SE. At the 0.05 significance level, the means in columns with the same letter are not significantly different. (**) It means highly significant differences between means in the same column.

**Table 5:** In both seasons of the study, the mean values of oil percent (%), oil content (mL plant^−1^, and oil yield (L ha^−1^) of jojoba shrubs as influenced by combined treatments of plant genotypes and foliar application of *Azolla filiculoides* Lam. plant extract.

Treatment		Oil Percent (%)		Oil Yield (mL plant^−1^)		Oil Yield (L ha^−1^)	
Jojoba Genotype	Azolla Extract (%)	1st Season	2nd Season	1st Season	2nd Season	1st Season	2nd Season
EAI1	Control	56.3 ± 3.2 a	57.1 ± 2.7 a	1258 ± 141 abc	1293 ± 121 b	1678 ± 188 abc	1725 ± 161 b
EAI1	10	53.7 ± 3.0 ab	55.0 ± 2.6 ab	1305 ± 146 ab	1368 ± 128 ab	1740 ± 195 ab	1824 ± 170 ab
EAI1	30	50.8 ± 2.9 bc	52.1 ± 2.5 bcd	1432 ± 160 a	1501 ± 140 a	1910 ± 214 a	2002 ± 187 a
EAI2	Control	48.9 ± 2.8 cd	49.6 ± 2.3 de	949 ± 106 e	976 ± 91 e	1265 ± 141 e	1301 ± 122 e
EAI2	10	46.2 ± 2.6 de	47.3 ± 2.2 ef	965 ± 108 e	1012 ± 95 de	1287 ± 144 e	1349 ± 126 de
EAI2	30	43.7 ± 2.5 e	44.8 ± 2.1 f	1059 ± 118 de	1110 ± 104 cde	1412 ± 158 de	1481 ± 138 cde
EAI3	Control	52.3 ± 3.0 bc	53.0 ± 2.5 bc	1083 ± 121 cde	1114 ± 104 cde	1444 ± 161 cde	1485 ± 139 cde
EAI3	10	48.8 ± 2.8 cd	50.0 ± 2.4 cde	1081 ± 121 cde	1133 ± 106 cd	1441 ± 161 cde	1511 ± 141 cd
EAI3	30	46.3 ± 2.6 de	47.4 ± 2.2 ef	1186 ± 133 bcd	1243 ± 116 bc	1581 ± 177 bcd	1658 ± 155 bc
F value		16.44 **	20.90 **	13.44 **	18.34 **	13.44 **	18.34 **

The data are expressed as a mean value ± SE. At the 0.05 significance level, the means in columns with the same letter are not significantly different. (**) It means highly significant differences between means in the same column.

**Table 6:** The relative percentage of fatty acids (%) of the fixed oil of jojoba oil as influenced by combined treatments of plant genotypes and foliar application of *Azolla filiculoides* Lam. plant extract.

Fatty Acids	The Relative Percentage of Fatty Acids (%)								
	EAI1			EAI2			EAI3		
	Control	10%	30%	Control	10%	30%	Control	10%	30%
Myristic Acid	2.05 ± 0.12	0.87 ± 0.05	1.51 ± 0.09	1.76 ± 0.10	0.75 ± 0.04	1.3 ± 0.07	1.86 ± 0.11	1.38 ± 0.08	0.79 ± 0.04
Myristoleic Acid	0.35 ± 0.02	1.37 ± 0.08	0.49 ± 0.03	0.3 ± 0.02	1.18 ± 0.07	0.43 ± 0.02	0.31 ± 0.02	0.45 ± 0.03	1.25 ± 0.07
Palmitic Acid	1.82 ± 0.10	1.3 ± 0.07	1.57 ± 0.09	1.57 ± 0.09	1.12 ± 0.06	1.35 ± 0.08	1.66 ± 0.09	1.43 ± 0.08	1.19 ± 0.07
Oleic Acid	11.76 ± 0.66	13.61 ± 0.77	12.39 ± 0.70	10.11 ± 0.57	11.71 ± 0.66	10.66 ± 0.60	10.7 ± 0.60	11.28 ± 0.64	12.39 ± 0.70
Linoleic Acid	1.42 ± 0.08	2.04 ± 0.12	1.68 ± 0.09	1.22 ± 0.07	1.76 ± 0.10	1.44 ± 0.08	1.29 ± 0.07	1.53 ± 0.09	1.86 ± 0.10
Gadoleic Acid	47.37 ± 2.67	49.83 ± 2.81	47.58 ± 2.69	43.11 ± 2.43	45.35 ± 2.56	43.3 ± 2.44	44.06 ± 2.49	44.25 ± 2.50	46.35 ± 2.62
Erucic Acid	13.35 ± 0.75	14.97 ± 0.85	13.72 ± 0.77	12.15 ± 0.69	13.63 ± 0.77	12.48 ± 0.70	12.42 ± 0.70	12.76 ± 0.72	13.93 ± 0.79
Nervonic Acid	12.26 ± 0.69	12.16 ± 0.69	12.96 ± 0.73	11.16 ± 0.63	11.07 ± 0.62	11.79 ± 0.67	11.41 ± 0.64	12.05 ± 0.68	11.31 ± 0.64
Total %	90.38	96.15	91.9	81.38	86.57	82.75	83.71	85.13	89.07
Saturated fatty acids	3.87	2.17	3.08	3.33	1.87	2.65	3.52	2.81	1.98
Unsaturated fatty acids	86.51	93.98	88.82	78.05	84.7	80.1	80.19	82.32	87.09

Data are mean value ± SE.

**Table 7:** In both seasons of the study, the mean values of chlorophyll a and b (mg g^−1^ FW) of jojoba shrubs as influenced by combined treatments of plant genotypes and foliar application of *Azolla filiculoides* Lam. plant extract.

Treatment		Chlorophyll a (mg g^−1^ **FW**)		Chlorophyll b (mg g^−1^ **FW**)	
Jojoba Genotype	Azola Extract (%)	1st Season	2nd Season	1st Season	2nd Season
EAI1	Control	0.853 ± 0.031 bc	0.888 ± 0.042 bc	0.403 ± 0.016 b	0.412 ± 0.027 bc
EAI1	10	0.890 ± 0.050 ab	0.911 ± 0.043 ab	0.418 ± 0.024 ab	0.428 ± 0.020 ab
EAI1	30	0.939 ± 0.053 a	0.962 ± 0.046 a	0.446 ± 0.025 a	0.457 ± 0.022 a
EAI2	Control	0.738 ± 0.028 e	0.762 ± 0.028 d	0.350 ± 0.010 c	0.358 ± 0.020 e
EAI2	10	0.765 ± 0.043 de	0.784 ± 0.037 d	0.360 ± 0.020 c	0.368 ± 0.017 de
EAI2	30	0.808 ± 0.046 cde	0.827 ± 0.039 cd	0.383 ± 0.022 bc	0.393 ± 0.019 cd
EAI3	Control	0.785 ± 0.032 cde	0.796 ± 0.037 d	0.381 ± 0.021 bc	0.390 ±0.018 cde
EAI3	10	0.810 ± 0.046 cd	0.829 ± 0.039 cd	0.401 ± 0.023 b	0.403 ± 0.019 bc
EAI3	30	0.855 ± 0.048 bc	0.875 ± 0.041 bc	0.406 ± 0.023 ab	0.415 ± 0.020 bc
F value		11.68 **	19.36 **	7.33 **	11.13 **

The data are expressed as a mean value ± SE. At the 0.05 significance level, the means in columns with the same letter are not significantly different. (**) It means highly significant differences between means in the same column.

**Table 8:** In both seasons of the study, the mean values of nitrogen (%), phosphorus (%), and potassium percentage (%) of jojoba shrubs as influenced by combined treatments of plant genotypes and foliar application of *Azolla filiculoides* Lam. plant extract.

Treatment		N (%)		P_2_O_5_ (%)		K_2_O (%)	
Jojoba Genotype	Azola Extract (%)	1st Season	2nd Season	1st Season	2nd Season	1st Season	2nd Season
EAI1	Control	2.79 ± 0.13 abc	2.86 ± 0.14 ab	0.375 ± 0.014 bcd	0.388 ± 0.018 bcd	2.60 ± 0.08 bc	2.62 ± 0.14 bc
EAI1	10	2.85 ± 0.16 ab	2.92 ± 0.14 ab	0.395 ± 0.022 ab	0.405 ± 0.019 b	2.69 ± 0.15 ab	2.76 ± 0.13 ab
EAI1	30	2.95 ± 0.17 a	3.03 ± 0.14 a	0.424 ± 0.024 a	0.434 ± 0.021 a	2.82 ± 0.16 a	2.89 ± 0.14 a
EAI2	Control	2.36 ± 0.10 f	2.43 ± 0.09 e	0.321 ± 0.009 f	0.337 ± 0.016 g	2.26 ± 0.07 f	2.33 ± 0.08 d
EAI2	10	2.46 ± 0.14 ef	2.51 ± 0.12 de	0.340 ± 0.019 ef	0.348 ± 0.016 fg	2.32 ± 0.13 ef	2.37 ± 0.11 d
EAI2	30	2.54 ± 0.14 def	2.60 ± 0.12 cde	0.364 ± 0.021 cde	0.373 ± 0.018 cde	2.42 ± 0.14 cdef	2.48 ± 0.12 cd
EAI3	Control	2.54 ± 0.09 def	2.58 ± 0.10 cde	0.347 ± 0.013 def	0.360 ± 0.017 efg	2.41 ± 0.06 def	2.45 ± 0.05 cd
EAI3	10	2.60 ± 0.15 cde	2.66 ± 0.13 cd	0.360 ± 0.020 cde	0.369 ± 0.017 def	2.45 ± 0.14 cde	2.51 ± 0.12 cd
EAI3	30	2.69 ± 0.15 bcd	2.75 ± 0.13 bc	0.386 ± 0.022 bc	0.395 ± 0.019 bc	2.56 ± 0.14 bcd	2.63 ± 0.12 bc
F value		11.94 **	17.17 **	16.71 **	21.43 **	13.07 **	11.69 **

The data are expressed as a mean value ± SE. At the 0.05 significance level, the means in columns with the same letter are not significantly different. (**) It means highly significant differences between means in the same column.

**Table 9:** The orchard soil physical and chemical analysis.

Parameters	Texture Class	ECe	Ca^++^	Mg^++^	Na^+^	K^+^	HCO3^−^	Cl^−^	SO4^2−^
		ds m^−1^	meq L^−1^						
Values	sandy	2.30	2.17	3.79	19.77	0.36	1.14	21.42	4.62

**Table 10:** Chemical properties of irrigation water used in this investigation.

Parameters	pH	EC	Ca^++^	Mg^++^	Na^+^	K^+^	HCO3^−^	Cl^−^	SO4^2−^
		ds m^−1^	meq L^−1^						
Values	7.42	3.46	5.39	3.70	21.94	3.32	1.98	29.51	3.26

**Table 11:** *Azolla filiculoides* Lam. plant extract chemical characteristics.

Parameters	Units	Values
Total flavonoid content	mg Rutin g^−1^ DW	24
Total phenolic content	mg Gallic g^−1^ DW	33
Protein	%	20.24
N	%	3.24
Mg	%	0.78
K_2_O	%	1.96
P_2_O_5_	%	0.32

**Table 12:** The combined treatments of jojoba genotypes and *Azolla filiculoides* Lam. plant extract.

Treatments	Jojoba Genotypes	Azolla Extract
T1	EAI1	0%
T2	EAI1	10%
T3	EAI1	30%
T4	EAI2	0%
T5	EAI2	10%
T6	EAI2	30%
T7	EAI3	0%
T8	EAI3	10%
T9	EAI3	30%

## Data Availability

The data presented in this study are available within the article.
